# Essential role of platelet activation via protease activated receptor 4 in tissue factor-initiated inflammation

**DOI:** 10.1186/ar2400

**Published:** 2008-04-15

**Authors:** Nathalie Busso, Veronique Chobaz-Péclat, Justin Hamilton, Pieter Spee, Nicolai Wagtmann, Alexander So

**Affiliations:** 1 Laboratoire de Rhumatologie, Centre Hospitalier Universitaire Vaudois, 1011 Lausanne, Switzerland; 2 Cardiovascular Research Institute, University of California at San Francisco, Parnassus Avenue, 94143, San Francisco, California, USA; 3 Monash University, Australian Centre for Blood Diseases, 89 Commercial Rd, Melbourne, Victoria 3004. Australia; 4 Biopharmaceuticals Biology, Novo Nordisk R&D, 2760 Bagsvaerd, Denmark

## Abstract

**Introduction:**

Tissue factor (TF) activation of the coagulation proteases enhances inflammation in animal models of arthritis and endotoxemia, but the mechanism of this effect is not yet fully understood – in particular, whether this is primarily due to fibrin formation or through activation of protease activated receptors (PARs).

**Methods:**

We induced extravascular inflammation by injection of recombinant soluble murine TF (sTF_1–219_) in the hind paw. The effects of thrombin inhibition, fibrinogen and platelet depletion were evaluated, as well as the effects of PAR deficiency using knockout mice deficient for each of the PARs.

**Results:**

Injection of soluble TF provoked a rapid onset of paw swelling. Inflammation was confirmed histologically and by increased serum IL-6 levels. Inflammation was significantly reduced by depletion of fibrinogen (*P *< 0.05) or platelets (*P *= 0.015), and by treatment with hirudin (*P *= 0.04) or an inhibitor of activated factor VII (*P *< 0.001) compared with controls. PAR-4-deficient mice exhibited significantly reduced paw swelling (*P *= 0.003). In contrast, a deficiency in either PAR-1, PAR-2 or PAR-3 did not affect the inflammatory response to soluble TF injection.

**Conclusion:**

Our results show that soluble TF induces acute inflammation through a thrombin-dependent pathway and both fibrin deposition and platelet activation are essential steps in this process. The activation of PAR-4 on platelets is crucial and the other PARs do not play a major role in soluble TF-induced inflammation.

## Introduction

The links between inflammation and coagulation have been the subject of intense research. On the one hand, inflammation activates the coagulation cascade and is prothrombotic; on the other hand, coagulation can also initiate and perpetuate inflammation. The molecules that are implicated in this cross-talk include tissue factor (TF), fibrin, the TF-generated coagulation proteases activated factor X and thrombin, and the protease activated receptors (PARs). In rheumatoid arthritis, we and other workers have shown that joint inflammation is accompanied by massive activation of coagulation proteases [1,2], and fibrin deposition can perpetuate inflammation in a murine model of RA [3]. Inhibition of thrombin activation and factor VII can also reduce synovial inflammation in these models [4,5].

TF is a glycoprotein that binds the serine protease activated factor VII (FVIIa) to initiate coagulation. Two major forms of TF are recognized; one cell bound, and the other in plasma or soluble form. Most of the known biological functions are attributed to the cell-bound form, but there are reports that soluble forms of TF may play a role in coagulation or hemostasis [6] and may be a link between tissue inflammation and thrombosis [7]. Soluble tissue factor (sTF) by itself can induce inflammatory arthritis when injected into mouse joints [8,9].

The precise mechanisms linking TF-dependent coagulation activation to extravascular inflammation are not fully understood. Thrombin activation of PAR-1 and PAR-4 can lead to G-protein-mediated cellular activation, as well as to NF-κB-mediated expression of P-selectin, E-selectin, vascular cell adhesion molecule 1 and intracellular adhesion molecule 1 adhesion molecules that favor leukocyte migration and activation in the vascular lining [10]. Fibrin, the final product of the coagulation cascade, can also be proinflammatory. Fibrin induces endothelial expression of adhesion molecules [11], and the fibrin degradation products are neutrophil chemotaxins [12]. Fibrin deposition in human glomerulonephritis and arthritis is associated with more severe disease [13,14]; in animal models of glomerulonephritis, arthritis and nerve injury fibrin exacerbates inflammation and tissue damage [3,15-17].

To assess the inflammatory effects of TF and the mechanisms involved, we studied the effects of TF when injected extravascularly. We showed that sTF injected into the mouse footpad is a potent proinflammatory stimulus and is critically dependent on both platelet PAR-4 and fibrin. These findings provide a better understanding of the role of TF activation in inflammation, and suggest potential targets for interrupting this pathway in disease states.

## Materials and methods

### Production of soluble tissue factor

sTF (residues 1 to 219 of murine TF) was expressed as inclusion bodies in *Escherichia coli*, harvested, and refolded essentially as previously described by Stone and colleagues [18] and Freskgard and colleagues [19]. Briefly, following expression, cells were harvested by centrifugation and resuspended in 100 ml of 50 mM Tris, 2 mM ethylenediamine tetraacetic acid, 0.1% Triton X-100, pH 8.0, and were lysed by sonication – after which, cell debris and inclusion bodies were recovered by centrifugation. The pellet was washed twice with 10 mM Tris, 1 mM ethylenediamine tetraacetic acid, 3% Tween 20, pH 7.5 and twice with H_2_O before it was dissolved in 6 M guanidine HCl, 50 mM Tris, 250 mM NaCl, pH 8.0. Refolding of the material was accomplished by dilution in 50 mM Tris, 250 mM NaCl, pH 8.5. The resulting solution was then concentrated and buffer exchanged into 20 mM Tris, 10 mM NaCl, pH 8.0 by diafiltration and then applied on a Q-sepharose ion-exchange column (Amersham Biosciences, Otelfingen Switzerland). The column was washed with 20 mM Tris, 20 mM NaCl, pH 8.0 and eluted using a 12-column volume gradient from 20 to 300 mM NaCl in 20 mM Tris, pH 8.0.

The resulting material was essentially pure at this point, as judged by SDS-PAGE and Coomassie staining. Endotoxin assay showed that the preparation was endotoxin free.

### Animals

PAR-1-deficient mice [20], PAR-2-deficient mice [21], PAR-3-deficient mice [22] and PAR-4-deficient mice [23] were bred from heterozygous mice, in a mixed Ola/C57Bl/6 background (PAR-1, PAR-3 and PAR-4 knockouts) or on a C57Bl/6 background (PAR-2, backcrossed >8 generations), and were used between 8 and 10 weeks old. Age-matched +/+ or +/- littermates were used as controls.

### Footpad inflammation

Ten microliters of sTF (0.2 to 5 μg/footpad) was administered into the intraplantar region of the right mouse hindfootpad. The contralateral footpad was injected with vehicle control (phosphate-buffered saline). Footpad swelling was evaluated using a caliper. Institutional approval was obtained for all animal experiments.

### Histological analysis

At least five mice per group were sacrificed, and the footpads were dissected and fixed in 10% buffered formalin for 7 days. Fixed tissues were decalcified for 3 weeks in 15% ethylenediamine tetraacetic acid, dehydrated and embedded in paraffin. Sagittal sections (8 μm) of the hind footpad were stained with Safranin-O and were counterstained with fast green/iron hematoxylin.

### Immunohistochemistry

Immunostaining was performed essentially as described elsewhere [3]. Fibrin immunostaining in the footpad was graded independently by two observers unaware of animal treatment on a scale of zero (no fibrin at all) to six (maximum of fibrin staining). Lymphocyte and macrophage infiltrations and endothelial cells in mouse synovium were detected using anti-CD3, anti-MAC-2, or anti-intracellular adhesion molecule antibodies, respectively, on paraffin-embedded sections as described previously [24].

### Thrombin–antithrombin III determination

The levels of thrombin–antithrombin III (TAT) complex in mouse plasma were measured by an ELISA kit designed for human TAT (Enzygnost TAT; Dade-Behring, Marburg, Germany), which cross-reacts with murine TAT. The content of murine TAT in plasma was calculated according to the human TAT standard curve.

### IL-6 measurements

Determination of IL-6 in serum was performed by ELISA (Amersham Biosciences, Otelfingen, Switzerland).

### Platelet depletion and platelet counts

Sixteen hours before injection with sTF, an intraperitoneal injection of 100 μl of 1/100 rabbit anti-mouse platelet serum (Accurate Chemicals, Westbury, NY, USA) was performed. Control mice received an injection of diluted normal rabbit serum. Platelet counts were performed using an automatic blood cell machine (Coulter Electronics, Miami, FL, USA). This dose of rabbit antimouse platelet serum resulted in >98% reduction in average number of circulating platelets after 24 hours and 40 hours.

### Anticoagulation treatments and systemic defibrinogenation

PEG-hirudin (Polyethylene glycol) (Knoll AG-BASF Pharma, Ludwigshafen, Germany) at 1 mg/kg was administered subcutaneously 1 hour before sTF injection. Active-site inhibited FVIIa (ASIS; Novo Nordisk, Bagsvaerd, Denmark) at 200 μg/kg was injected into the footpad just before sTF. Ancrod (Sigma Chemical Company, Buchs, Switzerland) at 100 U/kg was administered intraperitoneally

1 hour and 24 hours before injection with sTF, and resulted in >95% reduction in murine plasma fibrinogen as quantified by western blot. Equivalent amounts of phosphate-buffered saline were injected as control.

### Statistical analysis

Data are reported as mean values ± standard error of the mean. The Wilcoxon/Kruskal–Wallis (rank sum) test for unpaired variables was used to compare differences between groups with a non-Gaussian distribution. The unpaired Student's *t *test was used to compare groups with normally distributed values. All statistical calculations were performed using the JMP package (JMP version 4.02; SAS Institute, Cary NC 27513).

## Results

### Soluble tissue factor-induced footpad inflammation

**Figure 1:**
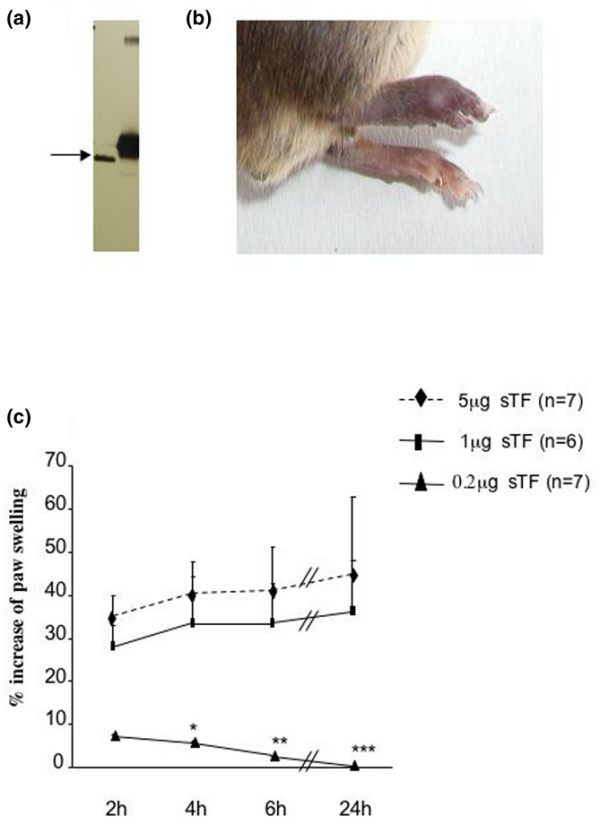
**Soluble tissue factor-induced footpad inflammation**. **(a) **Western blot of soluble tissue factor (sTF): 50 ng recombinant murine sTF (track 1) and 50 ng native TF (track 2) were detected using a polyclonal rabbit anti human TF antibody. **(b) **Effect of sTF injected into the footpad: 1 μg sTF (in 10 μl phosphate-buffered saline) was injected into the intraplantar region of the right hindfootpad. The contralateral footpad was injected with the same volume of phosphate-buffered saline. Swelling was observed in the sTF-injected footpad after 2 hours and was sustained over 24 hours (photograph). **(c) **Dose-dependent effect of sTF-induced inflammation: 0.2 to 5 μg sTF in 10 μl was administered into the hindfootpad. The contralateral footpad was injected with phosphate-buffered saline. Results expressed as the percentage increase in the right over left footpad thickness. *P < 0.05, **P < 0.01 and ***P < 0.001, Wilcoxon rank sum test.

We produced a recombinant form of TF, corresponding to the extracellular domain of murine TF (amino acids 1 to 219). The soluble recombinant protein migrated as a single band of ≈30 kDa (Figure 1a) and was purified as a single peak on mass spectrometry (data not shown).

Injection of sTF into the footpad of C57Bl/6 mice provoked an acute inflammatory response. Edema and erythema developed rapidly following injection (Figure 1b). The inflammatory response was quantified by measuring the footpad thickness. Paw swelling was maximal around 2 to 4 hours after injection (Figure 1c) and was sustained over 24 hours. Footpad swelling was dose dependent and the maximal effect was observed at 5 μg/injection. sTF blocked by prior incubation with inactivated FVIIa (ASIS; Novo Nordisk) did not induce footpad inflammation, thus confirming that it was sTF induced (data not shown). Serum IL-6 levels confirmed that inflammation was increased by sTF injection, and this was abrogated in ASIS-treated animals (Table 1).

**Table 1:** Plasma IL-6 levels after soluble tissue factor injection

	Untreated mice	Soluble tissue factor wildtype mice			
		
		No treatment	ASIS treatment	Hirudin treatment	Ancrod treatment
n	6	33	5	5	5
Mean (pg/ml)	<2	285	6.3	<2	<2
Standard error of the mean		107	6		

Plasma was collected 24 hours after soluble tissue factor footpad injection. In a parallel experiment, plasma was also collected from noninjected, naïve control mice. ASIS, active site inhibited activated factor VII.

**Figure 2:**
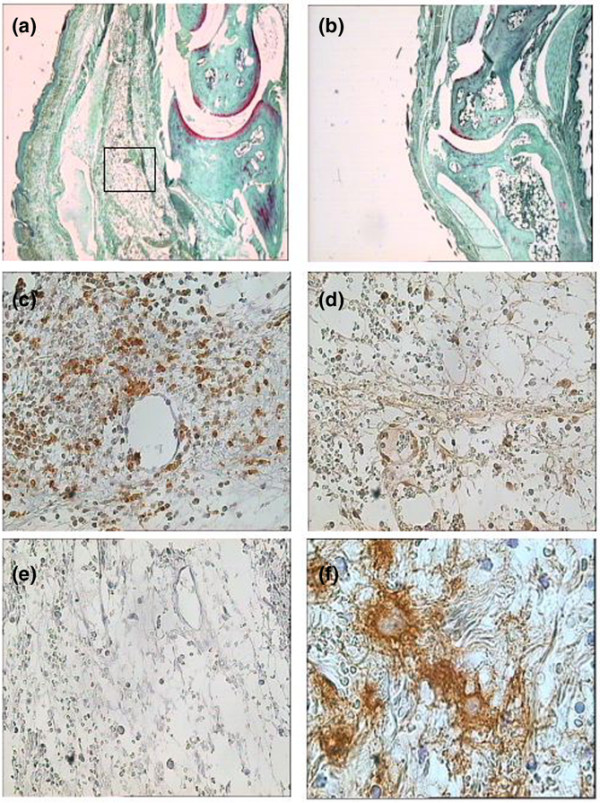
**Footpad histology and immunohistochemistry**. Samples were obtained 24 hours after soluble tissue factor injection. **(a) **Wildtype mice showed marked inflammatory changes. **(b) **Phosphate-buffered saline-injected mice showed minimal signs of inflammation. **(c) **Staining for macrophages was strongly positive. **(d) **CD3-positive T cells were also present. **(e) **Staining specificity was confirmed using, as primary antibody, nonimmune isotype-matched antibodies. **(f) **Fibrin deposition was assessed by fibrin immunohistochemistry.

Histological analysis showed pronounced edema and cellular infiltration (Figure 2a). Infiltrating inflammatory cells were predominantly macrophages (Figure 2c), with some CD3-positive T cells (Figure 2d). Fibrin staining was prominent (Figure 2f).

### Role of thrombin, factor VII and fibrin

**Figure 3:**
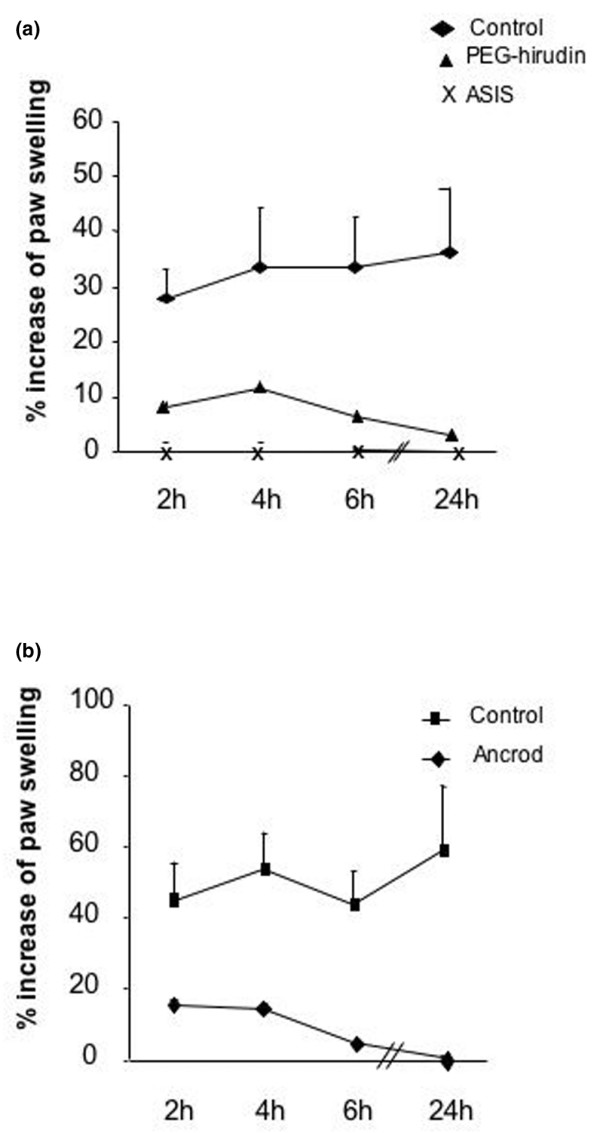
**Role of hirudin, factor VII inhibitor and ancrod in soluble tissue factor-induced footpad inflammation**. **(a) **Wildtype mice treated with PEG-hirudin (n = 5) or with ASIS (active-site inhibited activated factor VII, n = 5), or untreated mice (n = 7), were injected with 1 μg soluble tissue factor. Results from all treated groups were significantly reduced (*P *< 0.05 by *t *test) compared with the control group. **(b) **Wildtype mice were treated with ancrod (n = 7) or with phosphate-buffered saline (n = 7) and then injected with 1 μg soluble tissue factor. Results from ancrod-treated mice are significantly different from the control group, at all time points (*P *< 0.05 by *t *test).

We tested the effects of a specific thrombin inhibitor (PEG-hirudin), a FVIIa inhibitor (ASIS; Figure 3a), and a defibrinogenating agent (ancrod; Figure 3b) administrated prior to sTF injection in the same model. All treatments led to a marked reduction of footpad inflammation (*P *< 0.05 by *t *test for all time points with PEG-hirudin in comparison with wildtype control injected with phosphate-buffered saline; *P *< 0.001 for ASIS, *P *< 0.01 for ancrod). To assess that sTF was acting via FVIIa binding, we preincubated sTF *in vitro *with an excess of ASIS, hypothesizing that the preformed noncoagulant sTF–ASIS complex would not be able to induce coagulation upon its injection in the paw. As expected from the TF/FVIIa-dependent pathway, no inflammation of the paw was noticed upon injection of sTF–ASIS complex (sTF alone, 45 ± 17.5% versus sTF–ASIS complex, 0 ± 0% increase of paw swelling).

To evaluate the effect of these anticoagulation treatments on thrombin formation, we measured the plasma levels of TAT complexes in the different groups of mice. The TAT levels were increased in mice with sTF-injected footpads compared with sham-injected mice (sTF-injected mice, 31.55 ± 9.62 ng/ml; sham-injected mice, 8.28 ± 4.8 ng/ml). As expected, ASIS-treated and PEG-hirudin-treated mice showed reduced TAT levels (ASIS-treated mice, 8.2 ± 2.9 ng/ml; PEG-hirudin-treated mice, 14.3 ± 4.5 ng/ml). Serum IL-6 measurements confirmed the observed anti-inflammatory effect of the treatment administered (Table 1).

### PAR-4-deficient mice are resistant to soluble tissue factor-induced inflammation

**Figure 4:**
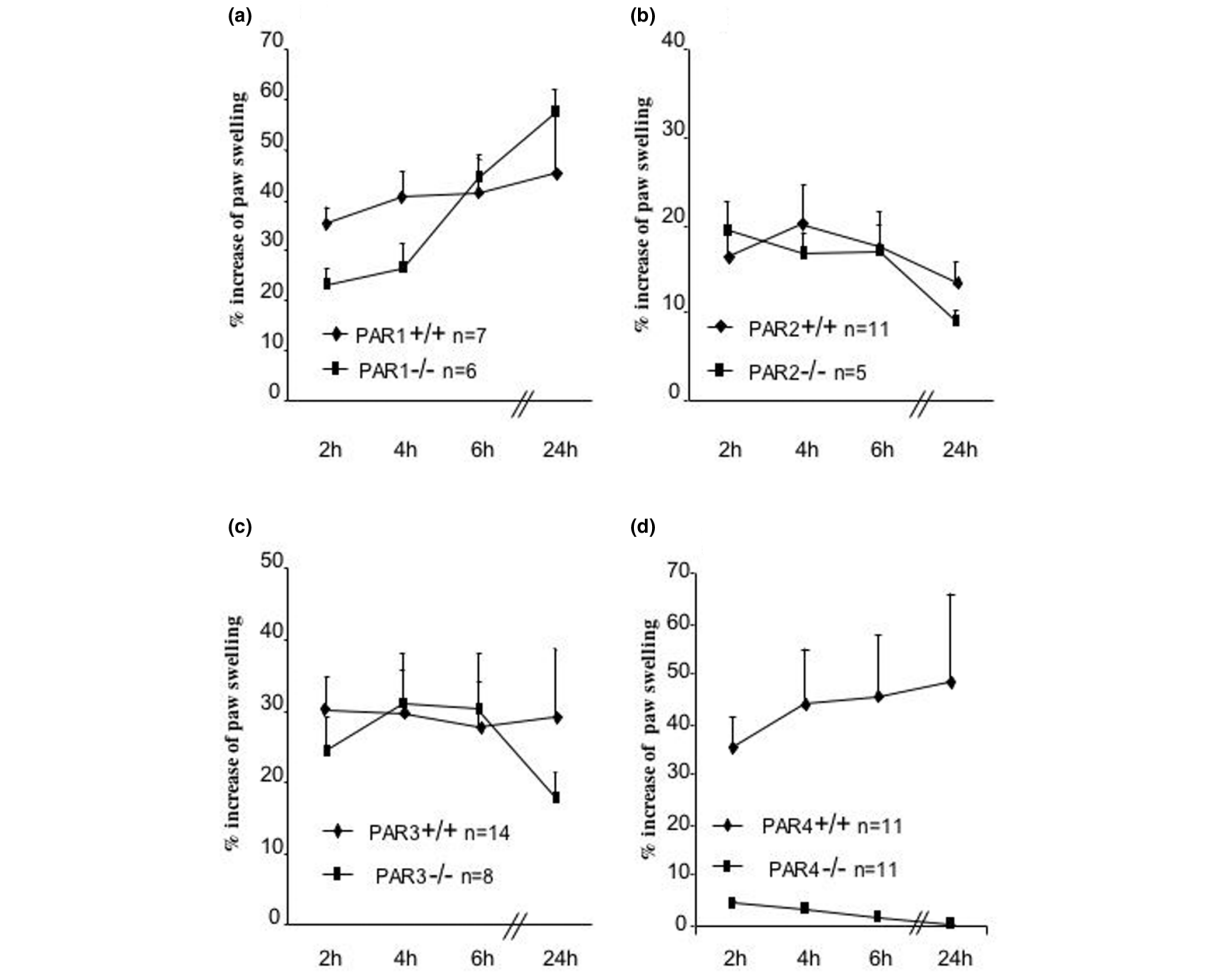
**Analysis of soluble tissue factor-induced inflammation in protease activated receptor-deficient mice**. Soluble tissue factor (1 μg) was injected into the footpad of mice with individual deficiency for one of the four different protease activated receptors (PARs). **(a) **PAR-1-deficient mice. **(b) **PAR-2-deficient mice. **(c) **PAR-3-deficient mice. **(d) **PAR-4-deficient mice. In each experiment, footpad swelling was assessed in the PAR-deficient mice and their littermates (+/+ or +/-) as controls.

To explore whether PARs play a role in sTF-induced footpad inflammation, we injected mice deficient for either PAR-1, PAR-2, PAR-3 or PAR-4. No differences were observed in footpad measurements between PAR-1-deficient, PAR-2-deficient and PAR-3-deficient mice when compared with their control littermates (+/+ or +/-) (Figure 4a to 4c). In contrast, PAR-4-deficient mice were almost totally resistant to sTF-induced inflammation compared with their littermates (Figure 4d).

**Figure 5:**
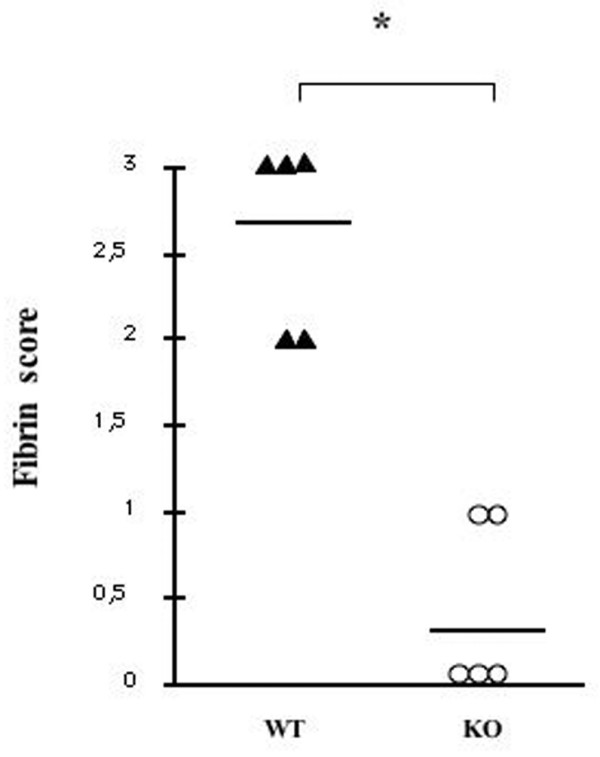
**Role of fibrin in soluble tissue factor-induced inflammation**. Fibrin immunohistochemical scores in protease activated receptor PAR-4^-/- ^mice (WT) compared with control mice (KO). *P = 0.001, Wilcoxon rank sum test. WT, wildtype; KO, knockout.

On histological analysis, PAR-4^-/- ^mice showed negligible signs of edema, hemorrhage and inflammation, and the histology was similar to that observed in control mice injected with vehicle alone (results not shown). We also examined fibrin deposition in the sTF-injected footpads. Scoring of fibrin deposition was significantly reduced in PAR-4^-/- ^mice compared with wildtype littermates (Figure 5).

### Role of platelets in soluble tissue factor-induced inflammation

**Figure 6:**
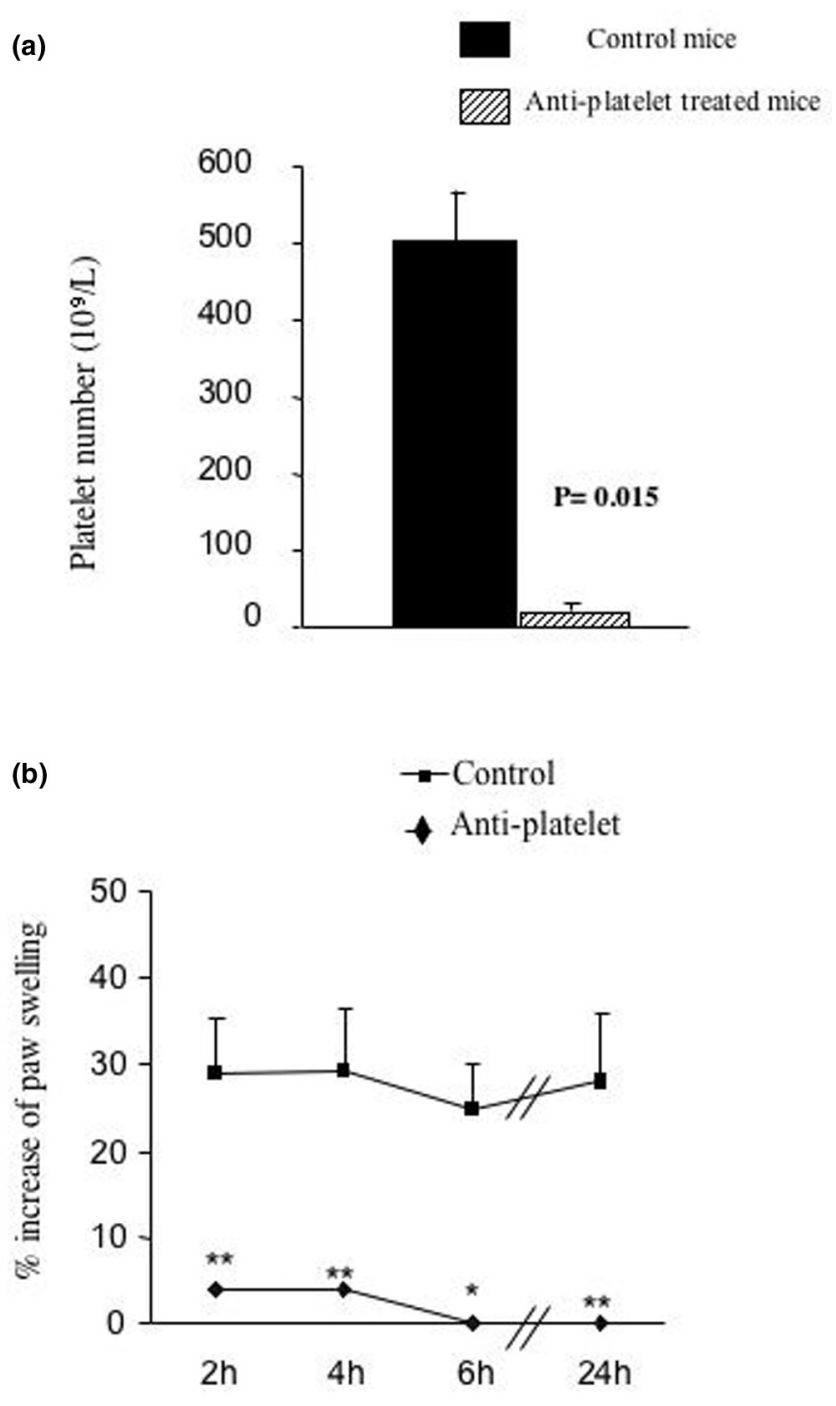
**Role of platelets in soluble tissue factor-induced footpad inflammation**. Immune thrombocytopenia was induced in protease activated receptor PAR-4^+/+ ^mice by a single antiplatelet antibody treatment given 16 hours prior to soluble tissue factor injection. **(a) **Platelet counts were performed 40 hours after injection of antibody. **(b) **Footpad swelling was greatly reduced in mice treated with antiplatelet antibody. Antiplatelet antibody-treated mice, n = 10; control, rabbit normal serum-treated mice, n = 10. *P < 0.05, **P < 0.01, *t *test.

As PAR-4 is predominantly expressed on platelets in mice [23], we investigated the contribution of platelets to sTF-induced inflammation. Thrombocytopenia was induced in wildtype mice by antiplatelet antibody treatment, resulting in a >98% reduction in the average number of circulating platelets (Figure 6a). The severity of inflammation was markedly reduced in mice treated with antiplatelet antibody, whereas sham-treated mice showed the usual footpad inflammation (Figure 6b). Histologic observations confirmed the reduction of footpad swelling in thrombocytopenic mice (results not shown).

## Discussion

Extravascular fibrin deposition is a hallmark of chronic inflammation and plays a role in perpetuating inflammation in rheumatoid arthritis, glomerulonephritis and experimental allergic encephalomyelitis [3,16,25]. TF-initiated coagulation accounts for fibrin formation and may also trigger inflammation through the action of downstream coagulation proteases such as thrombin and FVIIa on PARs. The relative roles of the different PARs in mediating inflammation are not well understood, however, and differing studies have implicated different PARs. We therefore chose to study the effects of sTF injected into the mouse footpad and the underlying mechanisms of its effects.

The injection of recombinant murine sTF into the mouse footpad results in an acute inflammation of extravascular tissues characterized by footpad swelling, histological signs of inflammation and fibrin deposition. This effect is mediated principally by the classical pathway of coagulation activation, through the formation of thrombin and fibrin, as inflammation was effectively blocked by administration of the thrombin inhibitor hirudin and an inhibitor of FVIIa. Depletion of fibrinogen by ancrod also attenuated inflammation in this model. These findings confirm that fibrin formation is an essential step in the link between coagulation and inflammation.

To determine whether PAR activation can also play a role in this model, we tested mice deficient for the individual PARs. To our surprise, only PAR4-deficient mice showed a phenotype, in that these mice were completely protected from sTF-induced inflammation. This contrasts with more chronic models that require an immune stimulus, such as glomerulonephritis and arthritis, in which PAR1 and PAR2 signaling seem to play a role [26,27]. Indeed, in antigen-induced arthritis, we found that TF/FVIIa activates PAR2 and subsequent arthritis – but in the same conditions, PAR4-deficient mice were indistinguishable from wildtype mice [28]. The key role of PAR4 suggested to us that platelet activation may be critical for inflammation to develop following sTF injection, as PAR-4 is the main platelet protease receptor in mice. This was confirmed when we performed the same experiments on normal mice that were rendered thrombocytopenic by the administration of antiplatelet antibody. The fact that PAR-3 had no effect at all on inflammation suggests that PAR-4 is the main pathway by which proteases activate mouse platelets *in vivo*.

One could question the physiological relevance of sTF in inflammation. Circulating forms of TF have been measured in different disease states [1,7], although it is likely to be much less abundant than cell-bound TF. There is much debate regarding what soluble TF consists of and whether it is biologically active [29]. A large proportion is probably in the form of microparticles but an alternatively spliced variant of natural TF has also been described [30,31]. Both forms have been reported to possess functional procoagulant activity [6,32], and increased levels of microparticular TF have been linked to vascular disease. The proinflammatory effects of the form of sTF we used in this study (containing amino acids 1 to 219) *in vivo *resembled those reported by Bokarewa and colleagues, who observed a chronic erosive arthritis after injection of a similar dose of human TF_1–219 _into mouse knees [8,9]. A difference is the time course, with an acute response in hours after injection in our hands, whereas in Bokarewa and colleagues' study, arthritis was observed from day 4 up to day 60 after injection.

Fibrin formation and deposition was significantly diminished in PAR-4-deficient mice and underlines the important role of fibrin in inflammation. These may be mediated by fibrin-induced macrophage secretion of proinflammatory cytokines such as IL-1β and TNFα [33-35] and of the chemokines MIP-1α, MIP-1β and MIP-2 [36]. The beneficial effect of fibrin depletion in different models of inflammation and neurodegenerative diseases reinforces the important role of fibrin deposition in perpetuating inflammation.

Finally, it is important to point out that both fibrin formation and platelet activation were needed for inflammation to develop in this model, and the absence of either of the two events abrogated inflammation. When PAR4^-/- ^mice were injected with sTF, there was an indication that coagulation was activated, as TAT levels were comparable with injected wildtype animals (PAR-4^+/+ ^mice, 33.2 ± 13 ng/ml, n = 16; PAR 4^-/- ^mice, 42.7 ± 20.8 ng/ml, n = 13). PAR-4-deficient mice did not show any clinical or histological signs of inflammation, however, and fibrin scoring was significantly lower. These findings indicate that the development of inflammation following coagulation activation requires the participation of platelets, probably via PAR-4 signaling, as well as the formation of fibrin. Activation of one pathway alone is insufficient to trigger full-blown inflammation. The results also suggest that blockade of either of the two arms of this process can prevent the development of inflammation after coagulation activation. Direct thrombin inhibitors such as hirudin could act on both fibrin formation and PAR activation simultaneously, and therefore should reduce coagulation-induced inflammation. This concept has yet to be tested in inflammatory diseases in man, although it has been shown to be effective in murine arthritis and ischemia models. Furthermore, our results would suggest that inhibitors of platelet PAR activation may also have a beneficial role in inflammation.

## Conclusion

Following subcutaneous injection of sTF in the mouse footpad, we observed both coagulation activation and acute inflammation. The inflammatory response required concomitant fibrin deposition and the participation of platelets. In this model, the platelet protease receptor PAR-4 plays a crucial role. In contrast, murine PAR-1, PAR-2 and PAR-3 did not have a major effect on inflammation. These results suggest that in chronic inflammatory diseases where fibrin deposition is significant, such as rheumatoid arthritis, inhibition of fibrin formation and platelet PAR can attenuate inflammation.

## Abbreviations

ELISA = enzyme-linked immunosorbent assay; FVIIa = activated factor VII; NF = nuclear factor; PAR = protease activated receptor; PEG = polyethylene glycol; sTF = soluble tissue factor; TAT = thrombin–antithrombin III; TF = tissue factor.

## Competing interests

The authors declare that they have no competing interests.

## Authors' contributions

NB and AS shared the design of the study and the writing of the manuscript. VC-P performed the experiments. JH provided the knockout mice used in the study. PS and NW generated the recombinant TF.

## References

[B1] So AK, Varisco PA, Kemkes-Matthes B, Herkenne-Morard C, Chobaz-Peclat V, Gerster JC, Busso N (2003). Arthritis is linked to local and systemic activation of coagulation and fibrinolysis pathways. J Thromb Haemost.

[B2] Carmassi F, De Negri F, Morale M, Song KY, Chung SI (1996). Fibrin degradation in the synovial fluid of rheumatoid arthritis patients: a model for extravascular fibrinolysis. Semin Thromb Hemost.

[B3] Busso N, Péclat V, van Ness K, Kolodziesczyk E, Degen J, Bugge T, So AK (1998). Exacerbation of antigen-induced arthritis in urokinase-deficient mice. J Clin Invest.

[B4] Marty I, Peclat V, Kirdaite G, Salvi R, So AK, Busso N (2001). Amelioration of collagen-induced arthritis by thrombin inhibition. J Clin Invest.

[B5] Busso N, Morard C, Salvi R, Peclat V, So A (2003). Role of the tissue factor pathway in synovial inflammation. Arthritis Rheum.

[B6] Szotowski B, Antoniak S, Poller W, Schultheiss HP, Rauch U (2005). Procoagulant soluble tissue factor is released from endothelial cells in response to inflammatory cytokines. Circ Res.

[B7] Sommeijer DW, Hansen HR, van Oerle R, Hamulyak K, van Zanten AP, Meesters E, Spronk HM, ten Cate H (2006). Soluble tissue factor is a candidate marker for progression of microvascular disease in patients with type 2 diabetes. J Thromb Haemost.

[B8] Bokarewa MI, Morrissey J, Tarkowski A (2002). Intra-articular tissue factor/factor VII complex induces chronic arthritis. Inflamm Res.

[B9] Bokarewa MI, Morrissey JH, Tarkowski A (2002). Tissue factor as a proinflammatory agent. Arthritis Res.

[B10] Minami T, Sugiyama A, Wu SQ, Abid R, Kodama T, Aird WC (2004). Thrombin and phenotypic modulation of the endothelium. Arterioscler Thromb Vasc Biol.

[B11] Qi J, Kreutzer DL, Piela-Smith TH (1997). Fibrin induction of ICAM-1 expression in human vascular endothelial cells. J Immunol.

[B12] Skogen WF, Senior RM, Griffin GL, Wilner GD (1988). Fibrinogen-derived peptide B beta 1–42 is a multidomained neutrophil chemoattractant. Blood.

[B13] Neale TJ, Tipping PG, Carson SD, Holdsworth SR (1988). Participation of cell-mediated immunity in deposition of fibrin in glomerulonephritis. Lancet.

[B14] Weinberg JB, Pippen AM, Greenberg CS (1991). Extravascular fibrin formation and dissolution in synovial tissue of patients with osteoarthritis and rheumatoid arthritis. Arthritis Rheum.

[B15] Akassoglou K, Adams RA, Bauer J, Mercado P, Tseveleki V, Lassmann H, Probert L, Strickland S (2004). Fibrin depletion decreases inflammation and delays the onset of demyelination in a tumor necrosis factor transgenic mouse model for multiple sclerosis. Proc Natl Acad Sci USA.

[B16] Drew AF, Tucker HL, Liu H, Witte DP, Degen JL, Tipping PG (2001). Crescentic glomerulonephritis is diminished in fibrinogen-deficient mice. Am J Physiol Renal Physiol.

[B17] Degen JL, Drew AF, Palumbo JS, Kombrinck KW, Bezerra JA, Danton MJ, Holmback K, Suh TT (2001). Genetic manipulation of fibrinogen and fibrinolysis in mice. Ann N Y Acad Sci.

[B18] Stone MJ, Ruf W, Miles DJ, Edgington TS, Wright PE (1995). Recombinant soluble human tissue factor secreted by *Saccharomyces cerevisiae *and refolded from *Escherichia coli *inclusion bodies: glycosylation of mutants, activity and physical characterization. Biochem J.

[B19] Freskgard PO, Olsen OH, Persson E (1996). Structural changes in factor VIIa induced by Ca^2+ ^and tissue factor studied using circular dichroism spectroscopy. Protein Sci.

[B20] Connolly AJ, Ishihara H, Kahn ML, Farese RV, Coughlin SR (1996). Role of the thrombin receptor in development and evidence for a second receptor. Nature.

[B21] Lindner JR, Kahn ML, Coughlin SR, Sambrano GR, Schauble E, Bernstein D, Foy D, Hafezi-Moghadam A, Ley K (2000). Delayed onset of inflammation in protease-activated receptor-2-deficient mice. J Immunol.

[B22] Kahn ML, Zheng YW, Huang W, Bigornia V, Zeng D, Moff S, Farese RV, Tam C, Coughlin SR (1998). A dual thrombin receptor system for platelet activation. Nature.

[B23] Sambrano GR, Weiss EJ, Zheng YW, Huang W, Coughlin SR (2001). Role of thrombin signalling in platelets in haemostasis and thrombosis. Nature.

[B24] Palmer G, Busso N, Aurrand-Lions M, Talabot-Ayer D, Chobaz-Peclat V, Zimmerli C, Hammel P, Imhof BA, Gabay C (2007). Expression and function of junctional adhesion molecule-C in human and experimental arthritis. Arthritis Res Ther.

[B25] Paul J, Strickland S, Melchor JP (2007). Fibrin deposition accelerates neurovascular damage and neuroinflammation in mouse models of Alzheimer's disease. J Exp Med.

[B26] Yang YH, Hall P, Little CB, Fosang AJ, Milenkovski G, Santos L, Xue J, Tipping P, Morand EF (2005). Reduction of arthritis severity in protease-activated receptor-deficient mice. Arthritis Rheum.

[B27] Cunningham MA, Rondeau E, Chen X, Coughlin SR, Holdsworth SR, Tipping PG (2000). Protease-activated receptor 1 mediates thrombin-dependent, cell-mediated renal inflammation in crescentic glomerulonephritis. J Exp Med.

[B28] Busso N, Frasnelli M, Feifel R, Cenni B, Steinhoff M, Hamilton J, So A (2007). Evaluation of protease-activated receptor 2 in murine models of arthritis. Arthritis Rheum.

[B29] Mackman N (2007). Alternatively spliced tissue factor – one cut too many?. Thromb Haemost.

[B30] Bogdanov VY, Balasubramanian V, Hathcock J, Vele O, Lieb M, Nemerson Y (2003). Alternatively spliced human tissue factor: a circulating, soluble, thrombogenic protein. Nat Med.

[B31] Bogdanov VY, Kirk RI, Miller C, Hathcock JJ, Vele S, Gazdoiu M, Nemerson Y, Taubman MB (2006). Identification and characterization of murine alternatively spliced tissue factor. J Thromb Haemost.

[B32] Nieuwland R, Berckmans RJ, Rotteveel-Eijkman RC, Maquelin KN, Roozendaal KJ, Jansen PG, ten Have K, Eijsman L, Hack CE, Sturk A (1997). Cell-derived microparticles generated in patients during cardiopulmonary bypass are highly procoagulant. Circulation.

[B33] Perez RL, Ritzenthaler JD, Roman J (1999). Transcriptional regulation of the interleukin-1β promoter via fibrinogen engagement of the CD18 integrin receptor. Am J Respir Cell Mol Biol.

[B34] Perez RL, Roman J (1995). Fibrin enhances the expression of IL-1β by human peripheral blood mononuclear cells. Implications in pulmonary inflammation. J Immunol.

[B35] Jensen T, Kierulf P, Sandset PM, Klingenberg O, Joo GB, Godal HC, Skjonsberg OH (2007). Fibrinogen and fibrin induce synthesis of proinflammatory cytokines from isolated peripheral blood mononuclear cells. Thromb Haemost.

[B36] Smiley ST, King JA, Hancock WW (2001). Fibrinogen stimulates macrophage chemokine secretion through toll-like receptor 4. J Immunol.

